# Excellent Tribological Properties of Lower Reduced Graphene Oxide Content Copper Composite by Using a One-Step Reduction Molecular-Level Mixing Process

**DOI:** 10.3390/ma11040600

**Published:** 2018-04-13

**Authors:** Haibin Nie, Licai Fu, Jiajun Zhu, Wulin Yang, Deyi Li, Lingping Zhou

**Affiliations:** 1 College of Material Science and Engineering, Hunan University, Changsha 410082, China; s1513w0876@hnu.edu.cn (H.N.); jiajun@hnu.edu.cn (J.Z.); hnuywl@hnu.edu.cn (W.Y.); lideyi@hnu.edu.cn (D.L.); 2 Hunan Province Key Laboratory for Spray Deposition Technology and Application, Changsha 410082, China

**Keywords:** copper, reduced graphene oxide, network structure, tribological properties

## Abstract

Reduced graphene oxide (RGO) composite copper matrix powders were fabricated successfully by using a modified molecular-level mixing (MLM) method. Divalent copper ions (Cu^2+^) were adsorbed in oxygen functional groups of graphene oxide (GO) as a precursor, then were reduced simultaneously by one step chemical reduction. RGO showed a distribution converting from a random to a three-dimensional network in the copper matrix when its content increased to above 1.0 wt.% The tribological tests indicated that the friction coefficient of the composite with 1.0 wt.% RGO decreased markedly from 0.6 to 0.07 at an applied load of 10 N, and the wear rate was about one-third of pure copper. The excellent tribological properties were attributed to a three-dimensional and uniform distribution, which contributes to improving toughness and adhesion strength.

## 1. Introduction

Wear failure is the most common mode in mechanical engineering. The development of advanced self-lubricating material is a potentially promising approach to improving the wear resistance. Cu-graphite composites have been widely researched for applications in electrical sliding contacts, such as brushes, bearing bushings and contact wires [1,2,3]. Generally, it is necessary to add a considerable amount of graphite to obtain good lubrication effects, which normally deteriorates the mechanical properties and electrical conductivity of the composite [3,4].

Graphene has been expected to replace graphite in composite Cu matrix composites due to its high strength, elasticity modulus [5,6,7], and lubricating properties [8,9]. As a reinforcement, it has been widely reported that graphene efficiently improves matrix mechanical property [10,11,12,13,14,15]. For example, the microhardness of electrodeposition of Cu-1.1 wt.% RGO is 151 HV, which is approximately 30% higher than copper [14]. The friction performance of Cu-graphene has also attracted a lot of attention [16,17,18]. Cu-graphene [19] shows a much lower friction coefficient and wear rate than Cu-graphite under the same volume fraction (from 2.5–10 vol.%). This could be ascribed to the formation of a compacted and stable solid lubricating film on the wear surface with graphene and copper. However, excess graphene could greatly degrade the performance of the copper composite, and it is difficult to form lubricating films on the exposed surfaces. Due to the chemical inertness between graphene and Cu, graphene tends to agglomerate in copper matrix [20]. It is still a challenge to disperse graphene in the Cu matrix composite homogeneously and with robust interfacial bonding. By considering good interfacial bonding, it is possible to improve frictional performance [3,21]. Hence, improving the adhesion strength and dispersion between graphene and Cu is key to obtaining high tribological performance.

A lot of methods have been investigated to improve the bonding strength between graphene and metal. Electroless plating was applied to improve the wettability of graphene. Compared with pure Cu, the composite with the addition of 0.5 wt.% graphene content exhibited an increase of 49.1% in yield strength [22]. Hexadecyl trimethyl ammonium bromide (CTAB) was also employed as a bonding agent to improve interface strength. Copper coated with CTAB with positive charges was absorbed by GO to achieve homogeneous dispersion, which was effectively able to improve the ultimate tensile strength and thermal conductivity of the Cu composite [23]. With an addition of only 0.3 wt.% RGO, the coefficient of friction improves significantly [20]. The addition of many reagents, however, not only complicates the procedure, but also introduces impurities. The MLM method is an effective and simple way that employs GO and copper acetate as a precursor for fabricating Cu-RGO composite [12]. The traditional MLM process involved two steps. Firstly, a NaOH solution is used to prevent reduction of GO before the formation of chemical bonds with Cu^2+^. Secondly, the dry mixture is subsequently reduced using hydrogen with high temperature. Copper oxide nanoparticles, which effectively hinder the aggregation of GO in the drying process. More importantly, the chemical bonding of GO and Cu^2+^ enhance the interfacial adhesion. Zhang et al. [24] fabricated Cu-RGO composites using modified MLM. 1.0 vol.% RGO was still uniformly dispersed in the matrix. Hwang et al. [12] prepared the Cu-RGO composite by using the MLM process. The elastic modulus and the yield strength of the 2.5 vol.% Cu-RGO composite were 131 GPa and 284 MPa, respectively, which is about 30% and 80% higher than for pure Cu. These reports show that graphene is able to be distributed homogeneously in the Cu-RGO composite using MLM, improving the mechanical properties.

In this study, in order to obtain excellent lubrication performance without weakening the mechanical properties of the copper composite, we have successfully synthesized Cu-RGO particles with lower RGO content using a one-step reduction with a modified MLM method, and the bulk Cu-RGO composites were fabricated using a vacuum hot-press sintering process. The distribution of graphene changed from a disordered distribution to a network structure with increasing RGO content. The effect of the graphene content on the microstructure and tribological properties was researched. Furthermore, the friction and wear mechanisms of the composites are discussed in-depth.

## 2. Experimental

### 2.1. Cu-RGO Composite Preparation

**Figure 1:**
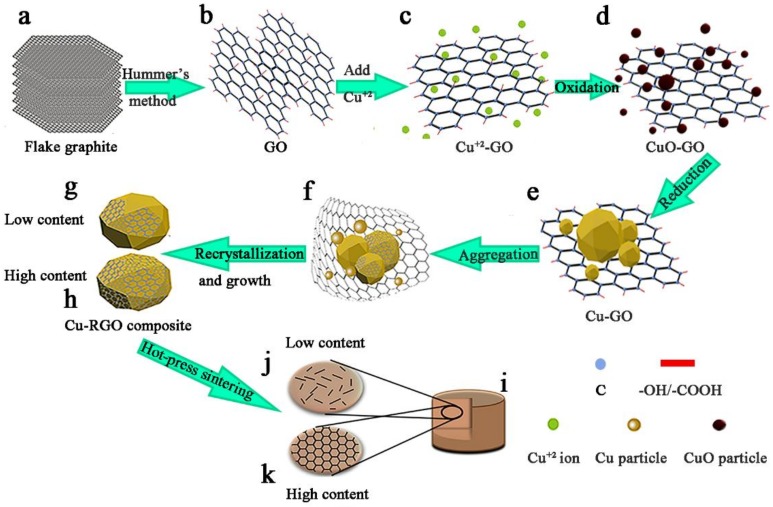
Schematic for the synthesis procedure of Cu-RGO composite: (**a**) flake graphite; (**b**) GO synthesized by Hummer’s method; (**c**) Cu^2+^ absorbed on the surface of GO; (**d**) oxidation of Cu^2+^; (**e**) Cu-GO obtained by reducing Cu^2+^ with ascorbic acid; (**f**) small Cu particles aggregated; (**g**,**h**) Cu-RGO power with low and high content of RGO; (**i**) bulk Cu-RGO composite consolidated by hot-press sintering; (**j**,**k**) spatial distribution of RGO in copper matrix.

The powders were prepared by a one-step reduction using a modified MLM method. Figure 1 shows the typical synthesis procedure of Cu-RGO composite. First, GO was prepared using a modified Hummers method [25]. Flake graphite (Figure 1a) was mixed with the mixture solution of H_2_SO_4_ (45 mL) and H_3_PO_4_ (5 mL) in an ice water bath. KMnO_4_ (7 g) was slowly added to the mixture under fast stirring, and the reaction was maintained below 20 °C for half an hour. After reaction at 50 °C for 10 h, the mixture was cooled to room temperature. After being diluted with deionized water, an appropriate volume of hydrogen peroxide was added under stirring until the solution turned golden and no bubbles came out. Finally, it was rinsed with 10 vol.% HCl solution to remove Mn^2+^ ions and was centrifuged with deionized water until the pH value approached 7.

Secondly, Cu-RGO powders were synthesized by a facile one-step reduction method. GO was diluted to a 0.5 mg/mL stable solution with deionized water by ultrasonic dispersion for 2 h. Then, aqueous GO was dropped into the solution of CuSO_4_·5H_2_O (1 M, 62.5 mL) and stirred vigorously to form Cu^2+^-GO solution (Figure 1c). After the mixture was heated to 90 °C without any gas, CuO-GO was formed with the addition of ammonia (Figure 1d). As a reducing agent, ascorbic (1 M, 75 mL) acid was added with vigorous stirring. CuO nanoparticles reduced to copper with aggregation and growth (Figure 1e,f). After being reduced for 3 h, Cu-RGO powders were formed (Figure 1g,h). Finally, the Cu-RGO powders were cleaned with deionized water several times and dried with a vacuum drying oven. The RGO content can be adjusted by the amount of GO.

Thirdly, the Cu-RGO powders were compacted by vacuum hot-press sintering (Figure 1j) at 900 °C for 30 min with a uniaxial pressure of 50 MPa. The final size of the consolidated Cu-RGO composite was 12.7 mm in diameter, with a thickness of 4 mm.

### 2.2. Material Characterizations

Tribological properties of Cu-RGO composites were characterized under ambient conditions (temperature of (20 ± 5) °C and relative humidity of 70~80%) by using a ball-on-disk machine (SFT-2M, Zhongke Kaihua Corporation, Lanzhou, China). The counterpart was a 52,100 steel ball with a diameter of 3 mm. The sliding speed was 0.1 m/s, with an applied load of 5 N or 10 N for 30 min. A super deep-scene 3D microscope (VHX-5000, Keyence, Osaka, Japan) was used to detect the worn traces in order to evaluate the wear volume V. The wear rate was characterized by W = V/s (s is total route). Every experimental parameter was tested at least three times.

Both the microstructure and composition of the wear tracks of Cu-RGO before and after wear tests were analyzed by SEM (QUANTA 200, FEI, Hillsboro, OR, USA) equipped with an energy dispersive X-ray spectrometer (EDS) (FEI, Hillsboro, OR, USA). Raman spectra of the wear tracks of Cu-RGO before and after the wear tests were also obtained at 1000 to 2000 cm^−1^ on a Labram-010 (Jobin Yvon, Paris, France) using a 632 nm laser. The chemical states and phase compositions inside and outside the wear tracks were analyzed by X-ray photoelectron spectroscopy (XPS-Thermo Fisher, K-Alpha 1063, Waltham, MA, USA). The analyzed spot size diameter was about 400 μm, and the thickness of the probed surface layer was approximately 10 nm. All XPS peaks were calibrated according to the C1s peak (284.8 eV). A Vickers microhardness tester (HVS-1000, Cai Kang Optical Instrument Corporation, Shanghai, China) was used to measure Cu-RGO under a load of 5 N ten times.

## 3. Results and Discussion

### 3.1. Microstructure of the Bulk Cu-RGO Composites

**Figure 2:**
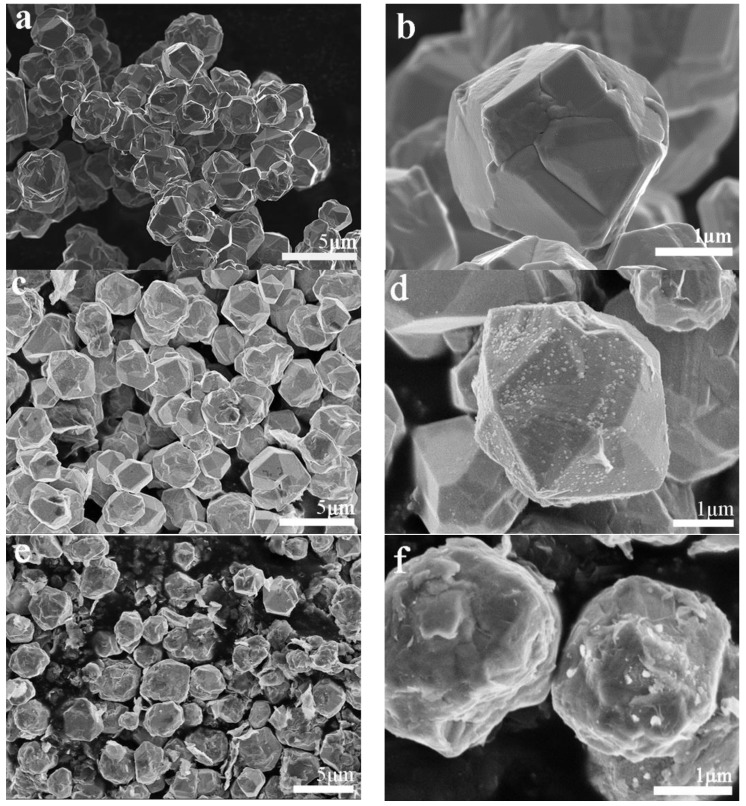
SEM images of (**a**) Cu; (**c**) Cu-0.2 wt.% RGO powders; (**e**) Cu-2.0 wt.% RGO powders; and magnified images of (**b**) Cu; (**d**) Cu-0.2 wt.% RGO powders; (**f**) Cu-2.0 wt.% RGO powders.

**Figure 3:**
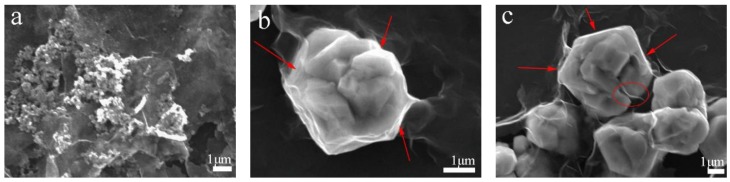
SEM images of Cu-2.0 wt.% RGO growing process: (**a**) heating to 90 °C; (**b**) after heating for 10 min; (**c**) after heating for 40 min.

Typical SEM images show the morphologies of the irregular polyhedral-shape Cu-RGO particles with sizes of 3–5 μm, as can be seen in Figure 2. Lots of the pure Cu particles are aggregated (Figure 2a), and the pure Cu particle surfaces are smooth and flat (Figure 2b). This aggregation gradually dissipates (Figure 2c) with the addition of 0.2 wt.% RGO. Some wrinkles and light points can be observed on copper particles (Figure 2d), which suggests that the RGO is coated onto the surfaces of the Cu particles. The Cu-RGO particles present more regularly, and the dispersion of the RGO increases to 2.0 wt.% (Figure 2e,f). In addition to the RGO coating, close observation shows that there is some RGO run-out from the Cu particles (Figure 2e). By examining the Cu-RGO particle growth process (Figure 3), it can be concluded that this may result from the one-step MLM process. The CuO nanoparticles, which were obtained by thermal decomposition of Cu(OH)_2_, are able to effectively reduce the aggregation of RGO (Figure 3a). After being reacted for 10 min at 90 °C, the particles coated by RGO gradually reduce to Cu and aggregate to micron particles. Due to the high surface energy, the layered RGO swirls around the aggregation (the red arrow in Figure 3b). As the duration of the heat treatment increases to 40 min, the particles further accumulate together to be about 3–5 μm in size (Figure 3c). Thus, RGO can be found both on the particle surface and internally (Figure 3c). After the reaction, the particles recrystallize and increase a little in size, with a final size of 3–5 μm (Figure 2e).

**Figure 4:**
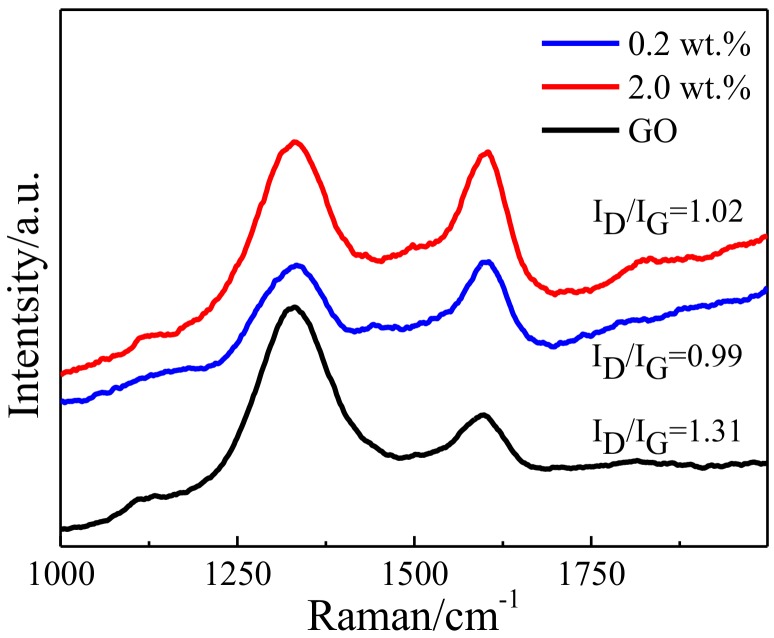
Raman spectra of GO and Cu-RGO powders.

**Figure 5:**
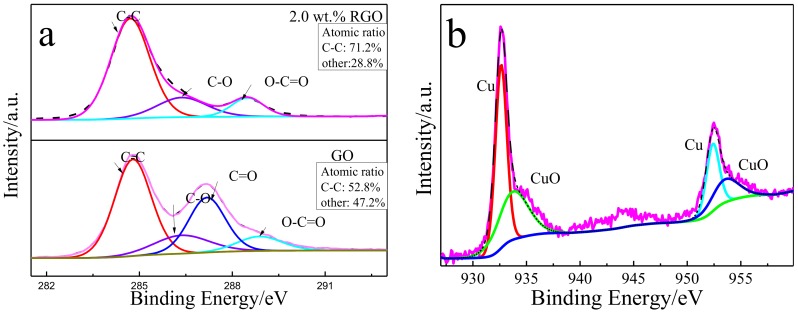
XPS spectra of C1s of (**a**) GO and Cu-RGO powders; (**b**) XPS spectra of Cu2p of Cu-2.0 wt.% RGO.

Raman spectra of Cu-RGO powders (Figure 4) show the well-known carbon D band (~1334 cm^−1^) and G band (~1600 cm^−1^). The I_D_/I_G_ ratio of Cu-RGO shown in Raman spectra is lower compared to that of pure GO, which confirms that GO is also reduced to RGO in this chemical reduction. Both the I_D_/I_G_ ratios of the composite powders are relatively high, which indicates higher defect density, which may result from the fact that ascorbic acid is a mild reducing agent, and the chemical bonding between RGO and Cu could damage the sp^2^ bonding network. The XPS spectra of C1s (Figure 5a) can be decomposed into four peaks comprised of sp^2^: C–C (284.7 eV), C–O (286.3 eV), C=O (287.8 eV) and O–C=O (288.5 eV) [8,26,27]. XPS spectra show that the contents of the oxygen-containing groups decrease from 47.2% for GO to 28.8% for Cu-RGO, suggesting that graphene oxide is partly reduced by ascorbic acid. It has been reported that the reaction between carboxyl or hydroxyl groups (O=C–OH, C=O, –OH) and copper atoms is able to produce Cu-oxygen bonds [28]. Wang et al. [29] found that the mechanical properties decrease with the decrease in oxygen-containing groups, due to the chemical bonding between Cu and RGO becoming weaker. Therefore, the 28.8% oxygen-containing groups in Cu-RGO may contribute to good bonding energy between Cu and RGO. This confirms the presence of RGO sheets in the Cu-RGO powders. The XPS spectra of Cu2p (Figure 5b) confirm the presence of CuO in the Cu particles. It is evidenced by the presence of the characteristic peaks of CuO at 933.7 eV and a broad shakeup satellite peak at 943.5 eV, combined with a side peak in the XPS spectra of Cu2p corresponding to CuO [30]. Broad satellite peak formation with the peak of CuO is obtained from the peak fit of the Cu2p_3/2_ spectrum. The oxidation state of Cu particles also confirms that RGO undergoes chemical boning with the Cu particles.

**Figure 6:**
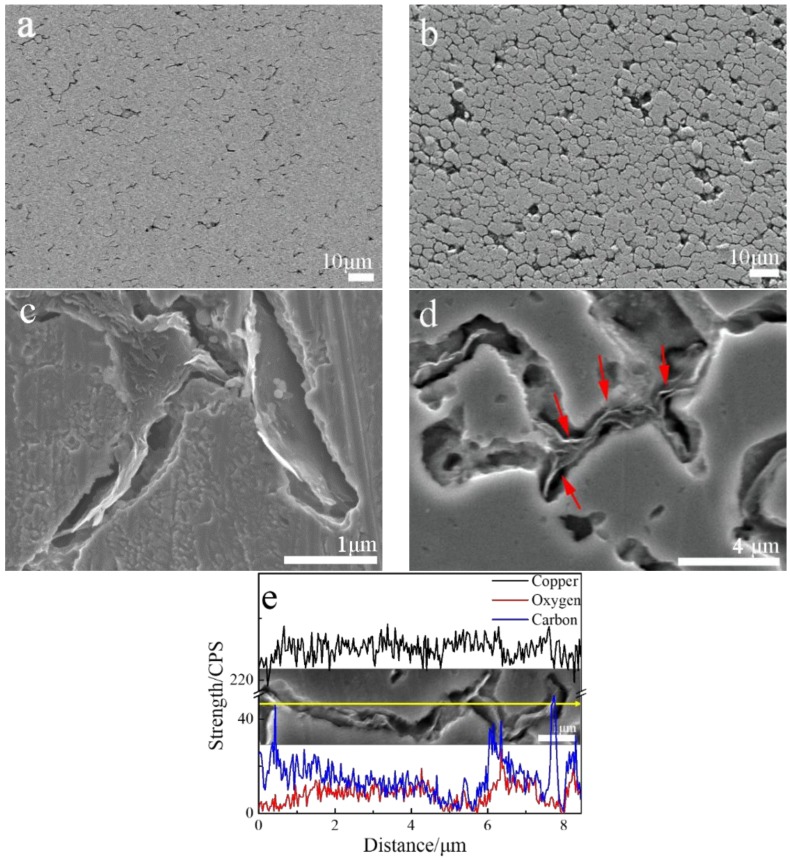
SEM images of etched cross-section of (**a**,**c**) Cu-0.2 wt.% RGO; (**b**,**d**) Cu-2.0 wt.% RGO; and (**e**) EDS of Cu-2.0 wt.%.

Figure 6 shows representative SEM images of the cross-section of the bulk Cu-RGO composites after being etched. The corrosion morphology exhibits a great difference between Figure 6a,b. A lot of the etch pit shows a disordered distribution in Cu-0.2 wt.% RGO, while it is connected to a net in Cu-2.0 wt.% RGO. The etch pits are magnified in Figure 6c,d. It is easy to find some RGO in the pit, which was further confirmed in the EDS line scanning image shown in Figure 6e. This indicates that the RGO was uniformly embedded in the Cu-RGO composite. Figure 6d shows that RGO is interconnecting in the etch pit, as indicated by the arrows, which suggests that RGO connects to a network structure. This three-dimensional structure increases the connection channel between RGO and RGO, which may improve the compressive capacity by the load transfer.

### 3.2. Tribological Properties of Cu/RGO Composites

**Figure 7:**
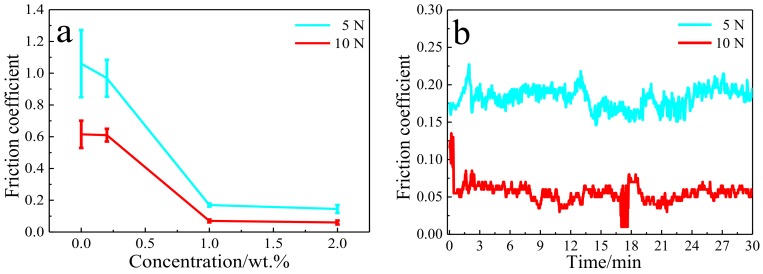
(**a**) Average friction coefficient of composites and (**b**) variation of friction coefficient of Cu-2.0 wt.% RGO as a function of sliding times under different applied loads.

The average friction coefficient (CoF) of Cu-RGO under different applied load is summarized in Figure 7a. With a little addition of RGO, the average CoF of Cu-0.2 wt.% RGO composite shows no obvious change compared to pure copper. However, the average CoF dramatically decreases to 0.07 when the RGO content increases to 1.0 wt.% (3.8 vol.%), under an applied load of 10 N. Previous studies have reported that the CoF of Cu-graphene is approximate 0.2, even though the graphene content is above 2.0 wt.% [14,19,31,32,33,34]. Chen et al. [31] used the MLM method to fabricate Cu-graphene; the CoF of a Cu composite with 4.0 vol.% graphene is still higher than 0.2. These results suggest that three-dimensional network structure composites show excellent lubricity with a lower content of RGO. When the content of RGO was further increased to 2.0 wt.%., the CoF did not change much. Another character is that the CoF of all Cu-RGO composites decreased as the applied load increased from 5 to 10 N. It may be that the lubricating film forms more easily under a load of 10 N than under that of 5 N. The CoF of Cu-2.0 wt.% RGO variation with sliding time describes the lubricating film status indirectly (Figure 7b). The CoF of Cu-2.0 wt.% RGO under 10 N is about 0.14 at initial step, that is close to 0.17 under 5 N. The films can be formed and broke between composite and counterpart during friction. This dynamic equilibrium is to be achieved, and the continuous lubricating film forms as the sliding time increases. Therefore, the CoF of Cu-2.0 wt.% RGO decreases to 0.06 and reaches a relatively steady status.

**Figure 8:**
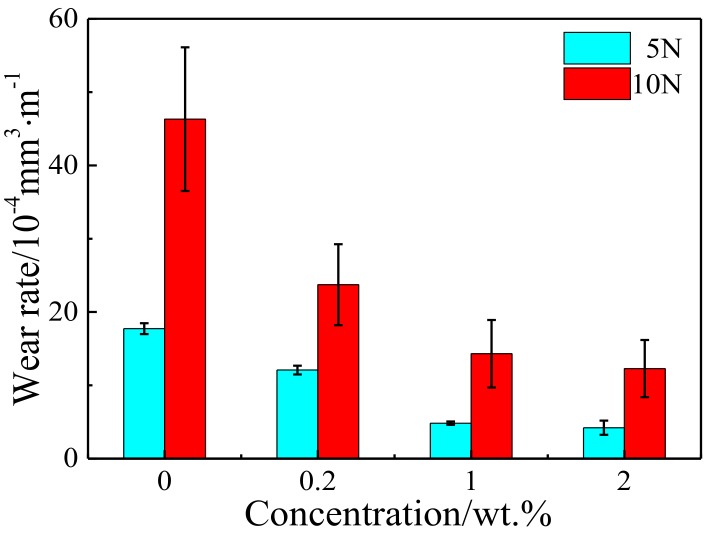
Wear rates of composites under different applied loads.

The variation of wear rates for Cu-RGO composites with applied loads are shown in Figure 8. Cu-1.0 wt.% RGO and Cu-2.0 wt.% RGO composites exhibited lower wear rates, not only than as-sintered pure Cu, but also at a level that was outstanding in the context of recent reports [32,34,35]. This can be attributed to the formation of a steady lubricating film, suggesting that addition of a certain content of RGO can significantly enhance the wear resistance. Despite the CoF of Cu-0.2 wt.%, RGO did not improved significantly compared with pure copper. However, it is easy to find that the wear rate of Cu-0.2 wt.% RGO is significantly lower than pure copper. This is owing to the enhancement of the hardness after a few RGO additions.

**Figure 9:**
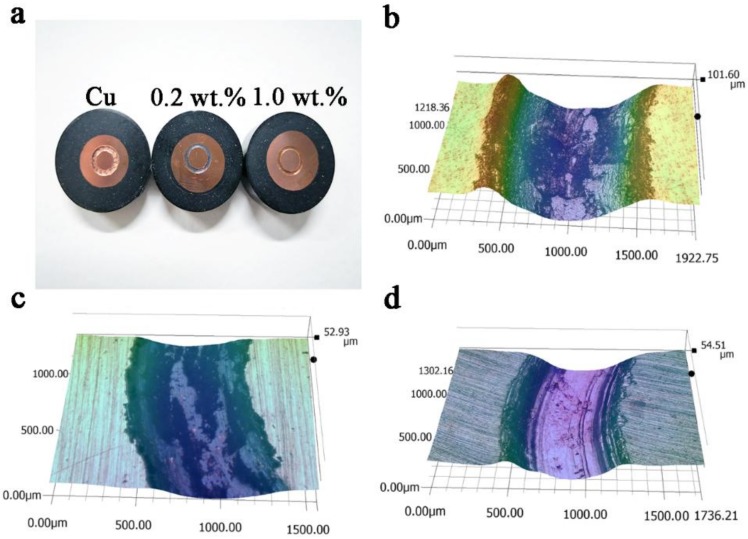
(**a**) Photograph of Cu and Cu-RGO composites, and 3D images of wear scars of Cu-RGO composites: (**b**) pure Cu; (**c**) Cu-0.2 wt.% RGO; (**d**) Cu-2.0 wt.% RGO after wearing under 5 N.

Figure 9 shows the photograph and 3D images of the wear scars of Cu and Cu-RGO composites. It is obvious that the depth and width of the wear scars decrease as the RGO content increases, corresponding to the wear rate. The wear scar of pure copper is very rough and irregular. After addition of RGO, the wear scars become smooth and flat. All the wear scars have a metallic luster except Cu-0.2 wt.% RGO in Figure 9a. It is easy to find that the oxygen content in Cu-0.2 wt.% RGO is much higher than that of the other materials (Table 1). This result demonstrates that Cu-0.2 wt.% RGO has severe oxidation during the friction and wear process, indicating that the metallic luster has been covered by a large amount of oxide. While it can only detect very low oxygen content in Cu-2.0 wt.% RGO, this is attributed to the formation of the continuous lubricating film, which avoids the direct contact between the composite and counterpart during friction and wear.

### 3.3. Friction and Wear Mechanism

**Figure 10:**
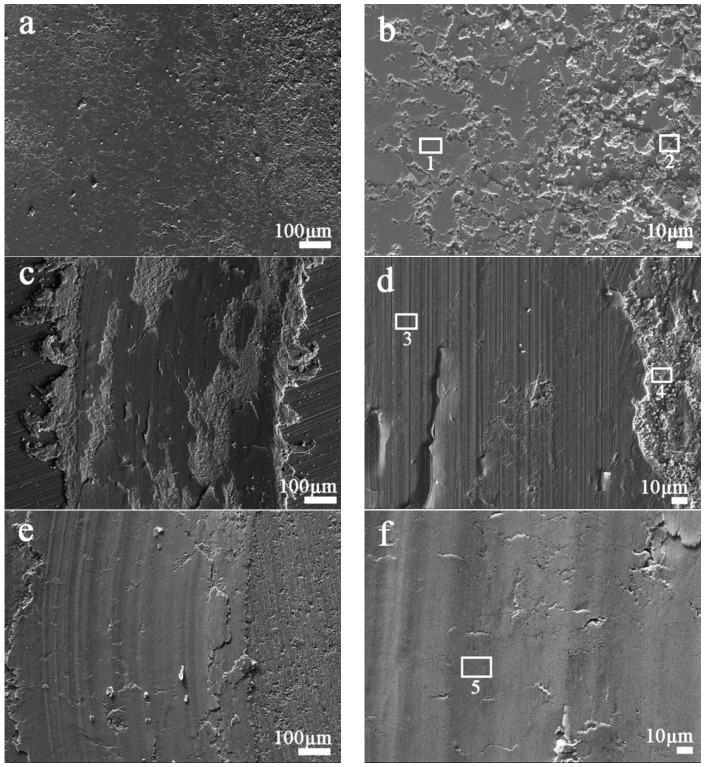
SEM morphologies of the wear tracks of (**a**,**b**) pure Cu; (**c**,**d**) Cu-0.2 wt.% RGO; (**e**,**f**) Cu-2.0 wt.% RGO after the sliding test under 5 N.

Figure 10a shows a great number of rupture symbols with fine debris on the worn surface under an applied load of 5 N. Since the ductility of Cu decreases with a little RGO addition due to the intermittent graphene distribution, more debris forms on the worn surface of the Cu-0.2 wt.% RGO (Figure 10c). The debris is oxidized easily during friction and wear. EDS analyses further confirm the oxidation phenomenon in Cu-0.2 wt.% RGO (Table 1) and debris entrapped in between the contact surfaces results in micro-cutting. It is clear from Figure 10d that the worn surface of the composite has a high order of roughness; additionally, deep wear grooves along the sliding direction are observed, which suggests the wear mechanism is dominated by abrasion. With further increases in the RGO content, the wear surface becomes smooth. No obvious fractures can be observed on the worn surface of Cu-2.0 wt.% RGO in Figure 10e. This may be attributed to the network structure, which creates a load transfer channel and acts as a whole to bear the compressive stress [36,37]. Rapid transfer of stress effectively avoids local stress that would be sufficiently high to cause surface fracture. Cu-RGO powders processed by the one-step MLM mixing process can effectively improve the bonding strength [12,24]. The delaminating scar and generation of cracks are found in Figure 10f. During sliding, plastic deformation occurs, resulting in an increase in wear rate, with a lot of RGO being exposed to the contact surface and compacted with metal debris to form a compacted protective film that has a self-lubricating ability and can partially avoid direct metal-to-metal contact. As a result, the lubricating properties of Cu-2.0 wt.% RGO composite are greatly improved.

**Figure 11:**
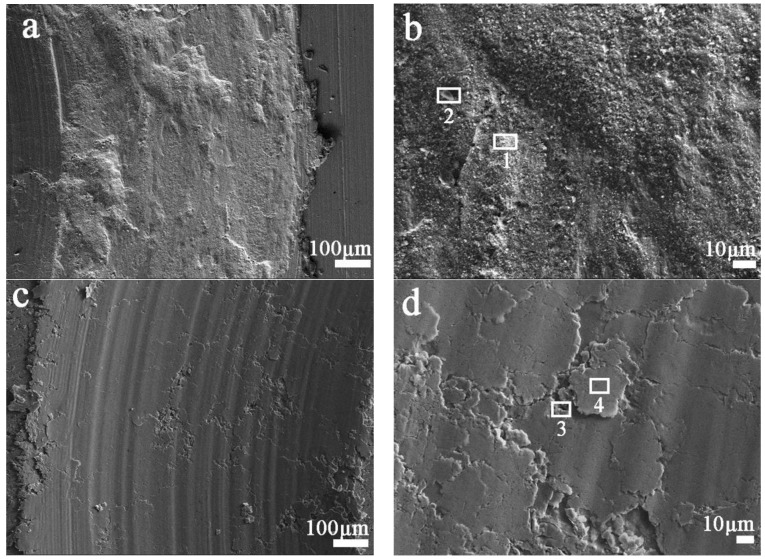
SEM morphologies of the wear tracks of (**a**,**b**) Cu-0.2 wt.% RGO; (**c**,**d**) Cu-2.0 wt.% RGO after the sliding test under 10 N.

As the applied load increases from 5 to 10 N, the worn surface fracture of Cu-0.2 wt.% RGO becomes more serious (Figure 11a), which results in an increase in the wear rate from 12.0 × 10^−4^ to 23.7 × 10^−4^ mm^3^·m^−1^. However, the worn surface still retains the smooth and continuous protective film of Cu-2.0 wt.% RGO, although it seems to show a large number of deep grinding cracks and furrows on the worn surface (Figure 11c). The local enlarged image (Figure 11d) shows that there are a few fractures and some debris. Table 2 shows that the carbon content of zone 4 is about 10.06 wt.%, which is far higher than Cu-0.2 wt.% RGO, while the oxygen content decreases to 1.26 wt.%. The Fe content of Cu-2.0 wt.% RGO is much lower than Cu-0.2 wt.% RGO, which comes from the 52,100 steel counterpart. This suggests that the protective film even contains carbon formed from graphene film on the worn surface of Cu-2.0 wt.% RGO, which avoids the oxidation of the worn surface.

**Figure 12:**
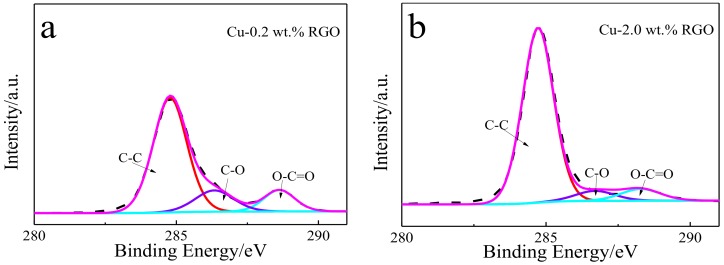
XPS spectra of C1s showing wear traces of Cu-RGO composites.

**Figure 13:**
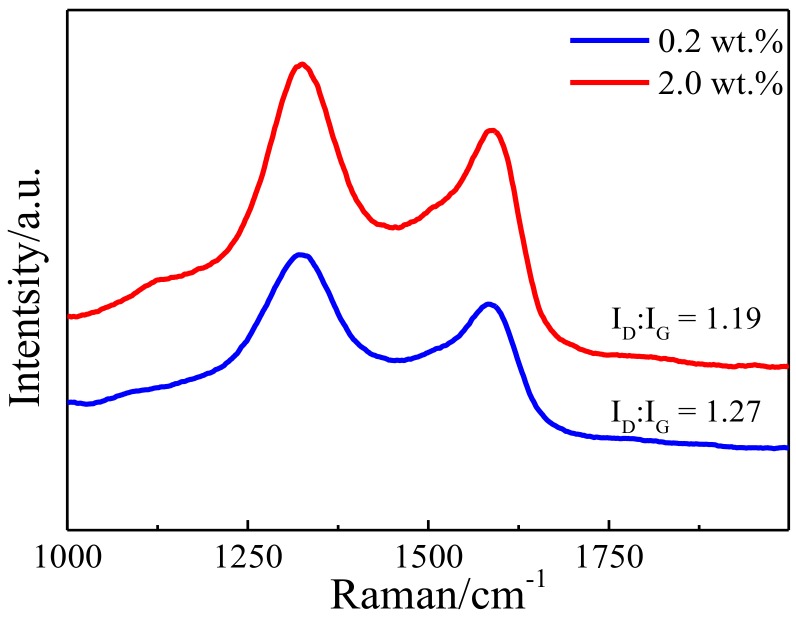
Raman spectra of wear traces of Cu-RGO composites.

As the applied load increases, more and more RGO is squeezed out of the composites and connects with the wear debris on the worn surface, forming well-consolidated graphene-rich films. This lubricating film is mainly affected by the content, spatial distribution, and size of the graphene particles [4]. When the RGO content is low, this is inadequate for the formation of continuous graphene film on the contact surface. Moreover, it is easy to destroy during friction. Although the XPS spectra of C1s (Figure 12a,b) confirm that a graphene lubricating film forms on the worn surface of Cu-0.2 wt.% RGO and Cu-2.0 wt.% RGO, the Raman spectra (Figure 13) shows the I_D_/I_G_ ratio of Cu-0.2 wt.% RGO and Cu-2.0 wt.% RGO after the wear test, suggesting that the RGO is damaged in the sliding process.

**Figure 14:**
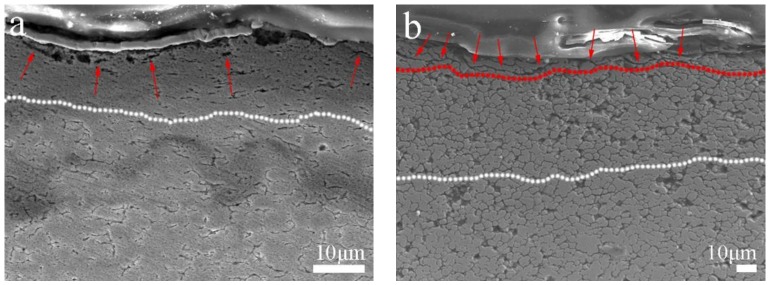
SEM of the etched cross-section after 5N friction test of (**a**) Cu-0.2 wt.% RGO and (**b**) Cu-2.0 wt.% RGO coated with a layer of tantalum as a protective layer.

**Figure 15:**
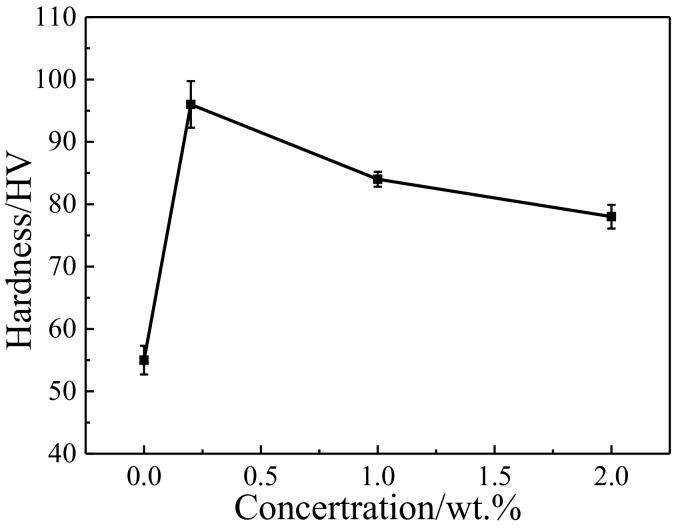
Hardness of Cu-RGO composites.

The depth of the deformation layer of Cu-0.2 wt.% RGO is about 10 μm, as shown in Figure 14a, which is lower than Cu-2.0 wt.% RGO due to the discontinuous RGO inducing weakness in the ductility of Cu-0.2 wt.% RGO. Therefore, the worn surface can be removed more easily than that of Cu-2.0 wt.% RGO. In the process of friction, lubrication films rupture and fall off constantly. The surface of RGO has been complemented and repaired, has made a solid lubricating film in the stage of dynamic balance, and has made CoF at a low and stable level. An obviously continuous RGO layer is exhibited on the worn trace of Cu-2.0 wt.% RGO, as shown with the red line (Figure 14b), but not Cu-0.2 wt.% RGO. This explains the unobvious decrease of CoF of Cu-0.2 wt.% RGO, while it decreases to below 0.07 for Cu-2.0 wt.% RGO. Plastic deformation made the three-dimensional structure more compact, which contributes to toughness enhancement, reducing the negative effect caused by stress concentration. Additionally, the formation of a lubrication membrane effectively improves the friction performance of the composite. Figure 15 shows the variation of hardness as the RGO content increases. Cu-0.2 wt.% RGO exhibits the highest hardness (97 HV), which is approximately 73% higher than pure Cu. This is attributed to the improvement in the dispersity and binding energy using MLM. The turning point appears at 1.0 wt.%., and the declining trend becomes slower, because the 3D structure improves the load transfer, which is beneficial to improving the wear resistance.

## 4. Conclusions

1. Cu-RGO composites were successfully prepared by one-step reduction with a modified MLM method and hot-press sintering. For such composites, the RGO distribution in the Cu-RGO composites shifts from unordered to three-dimensional as the RGO increases to above 1.0 wt.%.
2. There are benefits from this good, three-dimensional combination; the hardness of the composite with 2.0 wt.% RGO was without much deterioration, represented a 39% enhancement over pure Cu.
3. Compared to the as-sintered pure Cu, the Cu composite with only 1.0 wt.% RGO presented a lower friction coefficient and wear rates. The RGO improves the tribological behavior of copper matrix composites by hindering deformation of the copper matrix and forming a continuous lubrication transfer layer on the worn surface.

## Figures and Tables

**Table 1:** EDS analysis of Cu-RGO wear traces with various content of RGO after the sliding test under 5 N.

Zone	C (wt.%)	O (wt.%)	Fe (wt.%)	Cu (wt.%)
1	0	2.01	0.03	97.96
2	0	1.55	0.20	98.26
3	0	4.43	0.66	94.92
4	0	3.62	0.33	96.05
5	4.68	0.70	0.24	94.39

**Table 2:** EDS analysis of Cu-RGO wear traces with various content of RGO after the sliding test under 10 N.

Zone	C (wt.%)	O (wt.%)	Fe (wt.%)	Cu (wt.%)
1	2.99	6.28	1.22	89.52
2	3.74	4.46	0.87	90.92
3	16.99	1.48	0.00	81.52
4	10.06	1.26	0.21	88.47
